# Supplementary material for: Small extracellular vesicles reflect senescence progression in human bone marrow-derived mesenchymal stem cells during hollow fiber bioreactor culture
Source: Nanotheranostics. 2026 Jul 19;10:142–57. doi: 10.7150/ntno.132088 (PMC13540277; doi:10.7150/ntno.132088)
Supplement: Supplementary file 1 [file ntnov10p0142s1.pdf]

Supplementary information

Supplementary Methods (S1): Cartridge preparation: Prior to BMSC inoculation into the hollow fiber bioreactor system (HFBs), the cartridge was prepared according to the manufacturer's instructions. A polysulfone hollow fiber cartridge (catalog no. C2025D; surface area 450 cm²; extracapillary space (ECS) volume 3.2 mL; molecular weight cut-off 20 kDa was selected based on the desired extracellular vesicle (EV) yield and molecular size range. The cartridge was initially flushed and equilibrated with sterile phosphate-buffered saline (PBS) for 24 h. Subsequently, the polysulfone fibers were coated with fibronectin to enhance cell attachment by incubating the ECS with fibronectin (5–10 µg/mL; 100 µL fibronectin diluted in 3 mL PBS (without Ca/Mg) for 24 h at 37 °C. After incubation, the ECS was rinsed with 3 mL sterile PBS to remove unbound fibronectin. The cartridge was then sequentially conditioned with growth medium without fetal bovine serum (FBS) for 24 h, followed by complete growth medium for an additional 24 h prior to cell inoculation.

Supplementary Methods (S2): Inoculation of BMSCs into the Hollow Fiber Cartridge: BMSCs were inoculated into the hollow fiber system (HFS) at >95% confluency. Cells were detached using Accutase following removal of culture medium and washing with phosphate-buffered saline (PBS). The cell suspension was collected and centrifuged at 500 × g for 8 min at room temperature. The supernatant was discarded, and the cell pellet was resuspended in fresh culture medium. Cell viability and concentration were assessed prior to inoculation. Cells were resuspended in 3 mL of complete growth medium (10% FBS) and gently mixed to minimize aggregation. The hollow fiber cartridge was disinfected externally with 70% ethanol under sterile conditions. Sterile syringes were attached to the side ports, and the cell suspension was loaded into one syringe. Both side ports were opened, and the cell suspension was slowly injected into the ECS. To ensure homogeneous cell distribution, the suspension was gently pushed back and forth between syringes several times. After inoculation, the side ports were closed, flow through the cartridge was verified via the end ports, and the system was transferred to the incubator for culture.

Supplementary Methods (S3): HFB maintenance, harvesting, and medium adaptation:

High- glucose harvest protocol: the high-glucose harvest protocol was used for monitoring cell viability and metabolic activity and, when required, for EVs collection. The left end port was closed, and sterile syringes were attached to the side ports. With side ports opened, 2.5 mL of medium was withdrawn sequentially from each side port into the syringes. The collected medium was gently exchanged between syringes 3–5 times to ensure homogeneity, then transferred into a 15 mL conical tube. End ports were reopened, flow was verified, and the system was returned to the incubator.

Low-glucose harvest protocol: the low-glucose harvest protocol was applied for EV isolation and gradual fetal bovine serum (FBS) reduction. All ports were initially closed. A syringe containing 5 mL of fresh medium was attached to the left side port. With end ports closed, both side ports were opened, and medium was slowly infused from the left syringe while collecting the effluent into a syringe attached to the right-side port. The collected medium was transferred to a 15 mL conical tube. End ports were reopened, flow was verified, and the bioreactor was returned to the incubator.

Reservoir bottle medium exchange: medium exchange was performed every three days. The reservoir bottle was replaced with 175 mL of pre-warmed (DMEM or Xeno-free). Under sterile conditions, all ports were closed, the old medium was discarded, and fresh medium was added. End ports were reopened, flow was confirmed, and the system was returned to incubation.

Cell viability assessment: conditioned medium (3–4 mL) was collected using the high-glucose harvest protocol and centrifuged at 500 × g for 10 min at room temperature. The supernatant was retained for EV isolation. The cell pellet was resuspended in fresh medium, and viable cells were quantified using a cell counter. Cells were optionally replated in T25 flasks to assess viability and growth.

Adaptation to reduced FBS conditions: to minimize contamination with FBS-derived vesicles, BMSCs were gradually adapted to reduced FBS conditions using a combination of xeno-free medium and DMEM. Media were filtered (0.1

μm) prior to use. Due to the 20 kDa molecular weight cut-off of the hollow fiber membrane, soluble supplements were retained while serum-derived vesicles were excluded.

Supplementary Methods (S4): Extracellular vesicle (Small EV) isolation protocol: Conditioned medium (3–4 mL) was collected from the ECS using the low-glucose harvest protocol and subjected to sequential centrifugation. Samples were centrifuged at 400 × g for 10 min to remove cells, followed by 3,000 × g for 25 min to remove cellular debris. The supernatant was centrifuged at 7,000 × g for 25 min at 4 °C and subsequently at 18,000 × g for 20 min at 4 °C for further clarification. The resulting supernatant was ultracentrifuged at 150,000 × g for 90 min at 4 °C to pellet small extracellular vesicles. The sEV pellet was resuspended in filtered PBS and stored at −80 °C in PBS supplemented with 25 mM trehalose (Ca^{2+}/Mg^{2+}-free) until further analysis [1]

Supplementary Methods (S5): Cartridge cell removal and reconditioning protocol: To remove adherent cells and enable cartridge reuse, the ECS was filled with 5 mL Accutase and incubated at 37 °C for 30 min. The ECS and flow path were subsequently flushed thoroughly with PBS supplemented with penicillin–streptomycin and fungizone. The cartridge was sealed in a sterile storage bag and stored at 4 °C. Prior to reuse, the cartridge was reconditioned by replacing PBS in both the ECS and flow path with complete culture medium and perfused in the incubator at the maximum flow rate. Medium in the ECS and flow path was replaced daily for three consecutive days before reinoculation. Cells were then inoculated according to the standard operating procedure. Extended enzyme incubation (up to 2 h) and repeated flushing may be required to enhance cell detachment; however, complete removal of adherent cells cannot be guaranteed due to strong cell–fiber interactions. Detached cells were collected and cultured in T25 flasks to assess viability and potential contamination.

Supplementary Table (S1). Flow Cytometry panel configuration: Antibody–fluorochrome combinations, laser excitation, and detection channels used for phenotypic characterization of BMSCs under 2D and HFBS culture conditions.

marker	fluorochrome	excitation laser	detection channel	bandpass (nm)
CD105	PE-Cy7	488 nm	Ch1	773/56
CD90	PerCP-Cy5.5	488 nm	Ch6	702/87
CD73	PE	488 nm	Ch4	583/24
CD45	APC-Cy7	642 nm	Ch1	773/56
CD146	Alexa Fluor 647	642 nm	Ch6	702/87
CD271	PE-Cy7	488 nm	Ch1	773/56

Supplementary Table (S2). Compensation Matrix: Fluorescence spillover percentages applied for spectral overlap correction between fluorochromes prior to gating and histogram analysis:

	CD105	CD73	CD45	CD146	CD271
CD105	100	4.04	40.63	40.63	100
CD73	21.92	100	117.87	117.87	128.63
CD45	15.62	0.04	100	100	47.10
CD146	4.44	0.08	25.94	100	23.77
CD271	100	4.04	40.63	40.63	100

I) Antigen expression of normal BMSCs

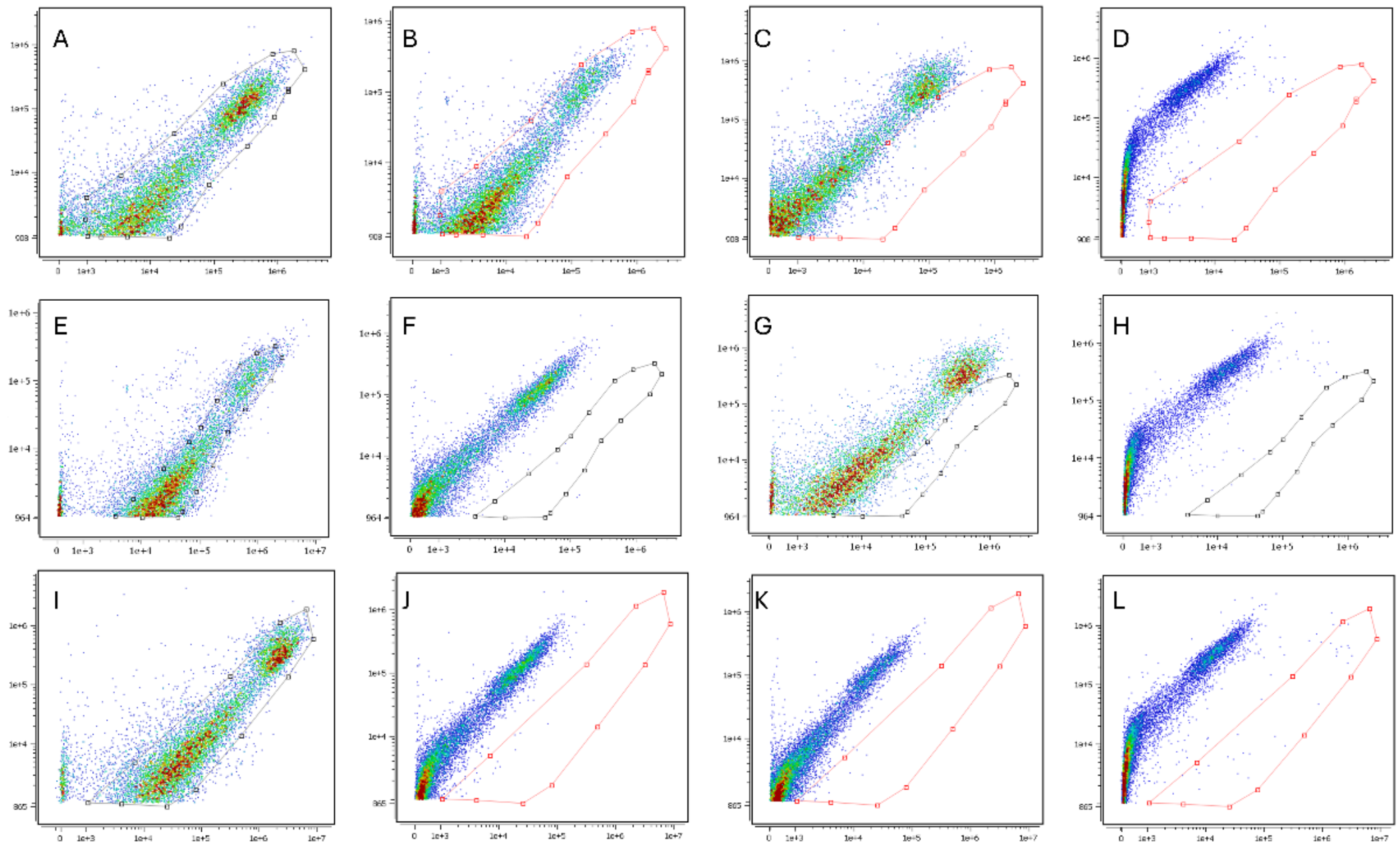

II) Antigen expression of senescence BMSCs

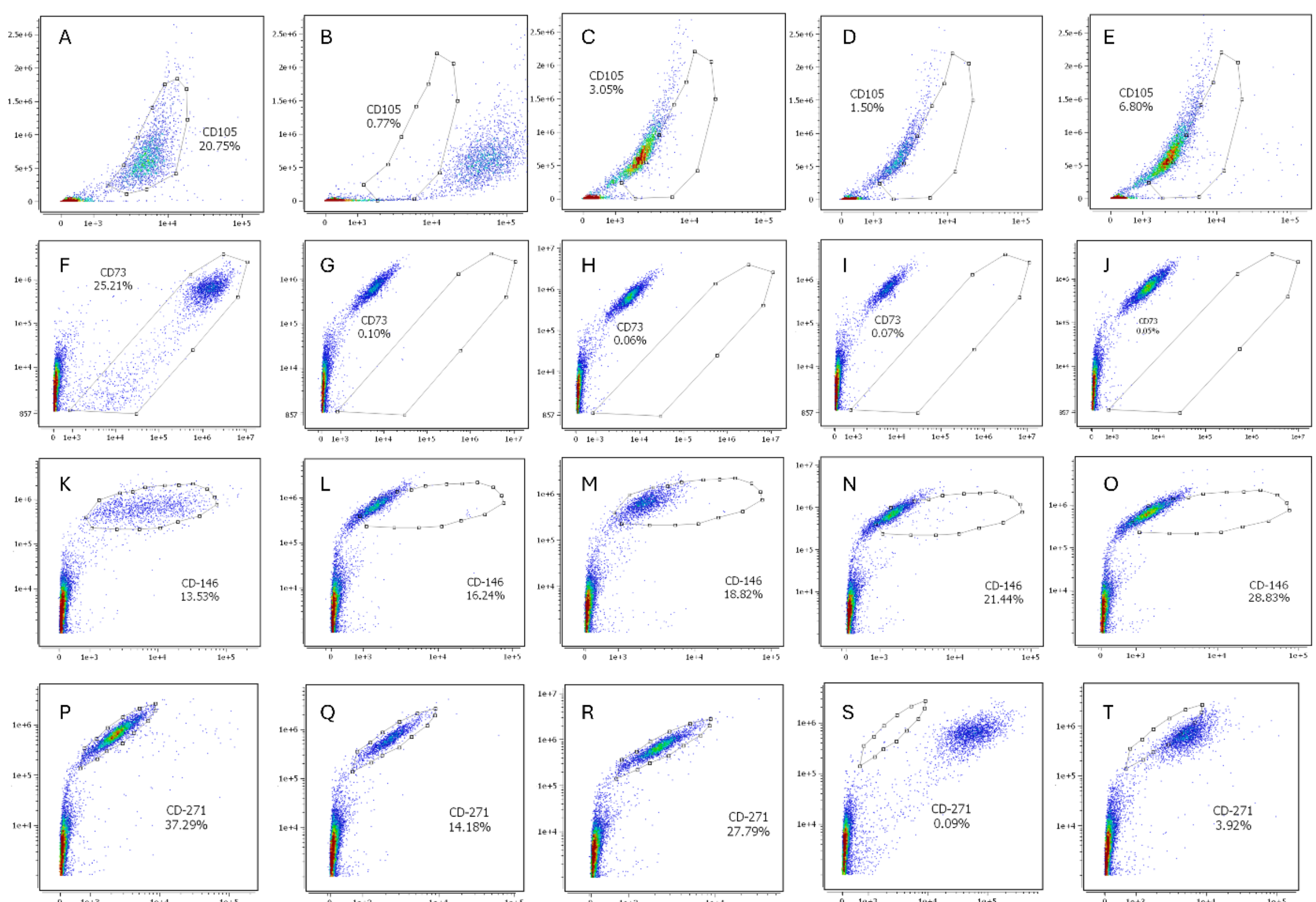

Supplementary Figures (S1): I) antigen expression of normal BMSCs (A–L): Representative gating strategy based on forward scatter (FSC) and side scatter (SSC) for BMSCs cultured under conventional 2D condition, showing selection of the population of interest and analysis of MSC markers CD105, CD90, and CD73. A, E, I) represent the results of gating with specific channels for CD105, CD90, and CD73 respectively. B, C, F, G, J, K) represent gating with unspecific chancels for regarding CD105, CD90, and CD73 antigens. D, H, L) represent unstained cells population regarding CD105, CD90, and CD73 antigens respectively. II) antigen expression of senescence BMSCs (A-T): Representative the same gating strategy for BMSCs cultured under 3D condition (HFBs), showing selection of the population of interest and analysis of MSC markers CD105, CD73, CD146 and CD271. In this case, A, F, K, P) represent the results of gating with specific channels for CD105, CD73, CD146 and CD271respectively. B, C, D, G, H, I, L, M, N, Q, R, S) represent gating with unspecific chancels for CD105, CD73, CD146 and CD271 antigens. Finaly, E, J, O, T) represent unstained cells population regarding CD105, CD73, CD146 and CD271 antigens respectively.

Supplementary Table (S3): Twenty-three samples were analyzed to assess the concentration and protein content of sEVs. The results showed a smooth correlation between sEVs population and their protein content

Stage	ROW	Sample Volume	NTA		Protein concentration (µg/ml): micro–BCA Protein Assay Kit
			sEVs population	Size (nm)	
phase1	1	100	4E+09	132	963.9
	2	100	3.55E+09	134	431.2
	3	100	3.23E+09	138	511.2
phase 2	4	140	2.85E+09	166	996.4
	5	180	4.11E+08	192	361.2
	6	250	3.52E+09	198	598.7
	7	300	5.38E+09	167	598.9
	8	200	1.07E+09	186	448.7
	9	200	9.37E+08	128	244.4
	10	170	1.26E+09	170	527.5
	11	150	3.97E+08	174	287
phase 3	12	140	3.72E+08	161	306.7
	13	500	8.24E+09	196	897.7
	14	300	3.13E+09	161	135. 3
	15	250	3.44E+09	201	303.7
	16	140	9.98E+08	147	203.7
	17	150	1.08E+09	140	265.3
	18	200	2.06E+09	165	1108.0
	19	250	1.01E+09	195	218.7
	20	150	3.35E+09	143	632
	21	200	3.7E+09	150	912.8
	22	140	2.38E+09	155	411.2
	23	140	9.01E+08	178	510.3

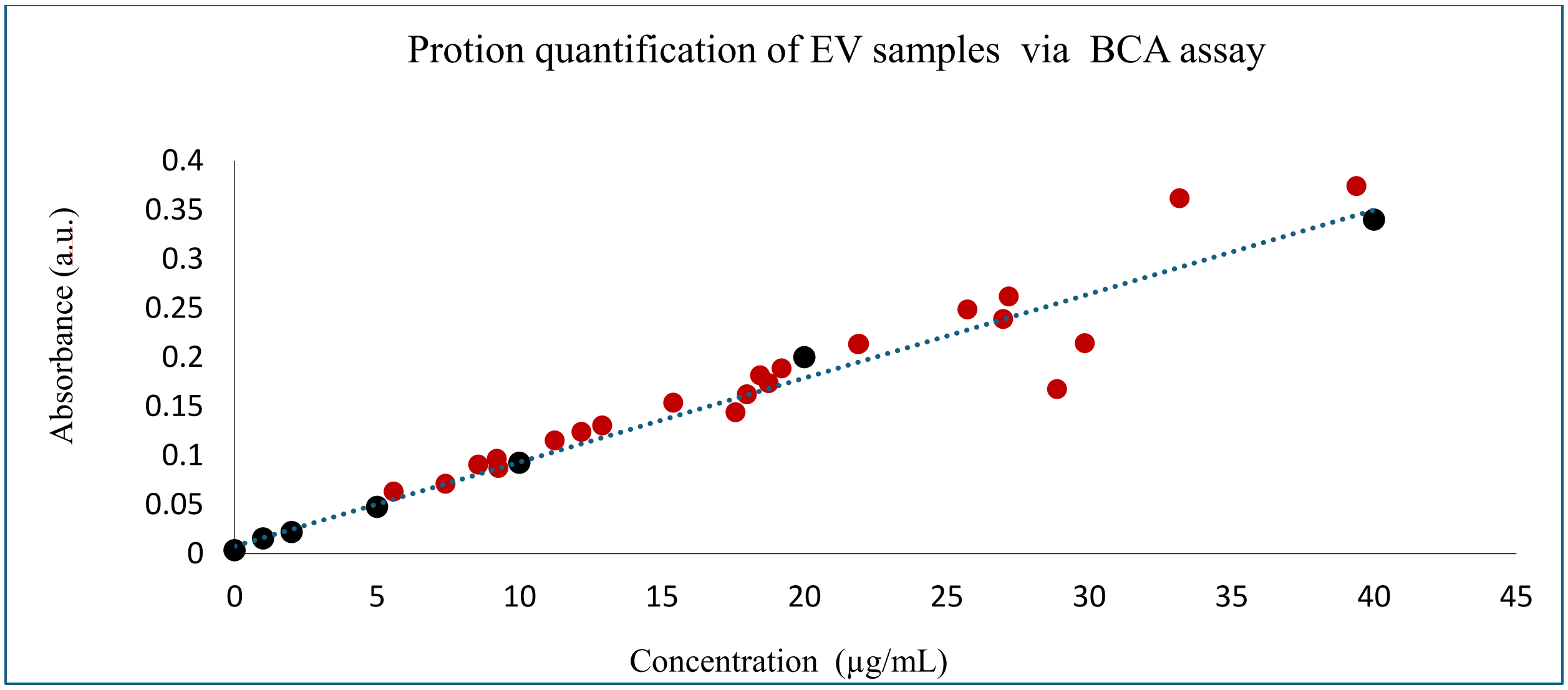

Supplementary Figures (S2): Protein quantity of sEVs isolated from the HFB system. Total protein content of sEV preparations was determined using the micro-BCA assay across 23 independent samples, providing a quantitative assessment of vesicle-associated protein yield

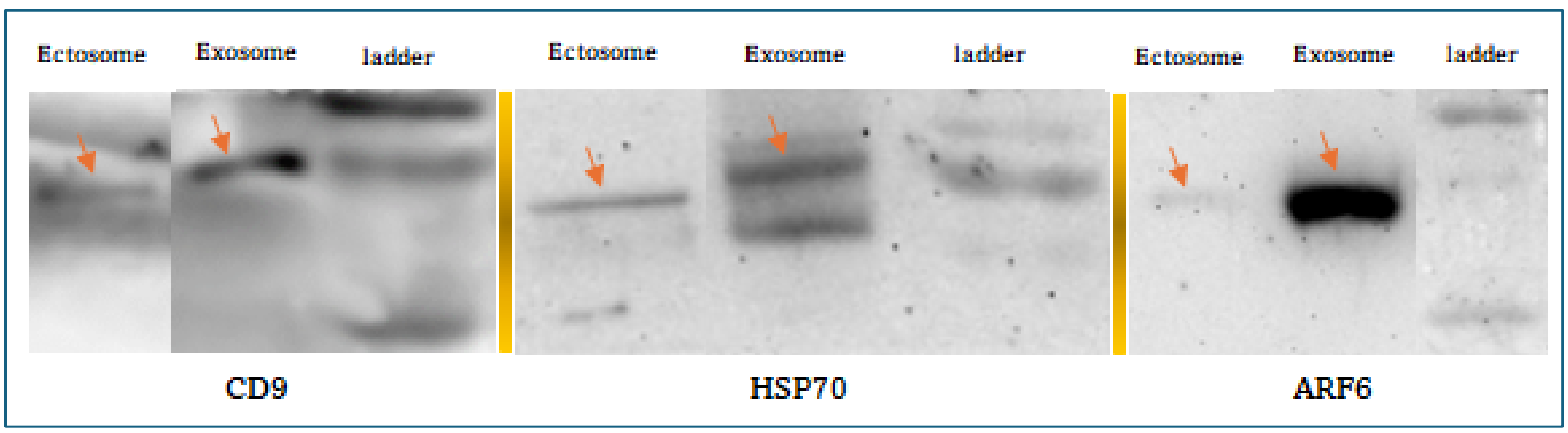

Supplementary Figures (S3): Western blot analysis of MSC-derived EVs, showing different expression of exosome and ectosome markers. Isolated EVs (exosome and ectosome-enriched fractions) were probed for CD9 and SP70, and ARF6 markers separately.

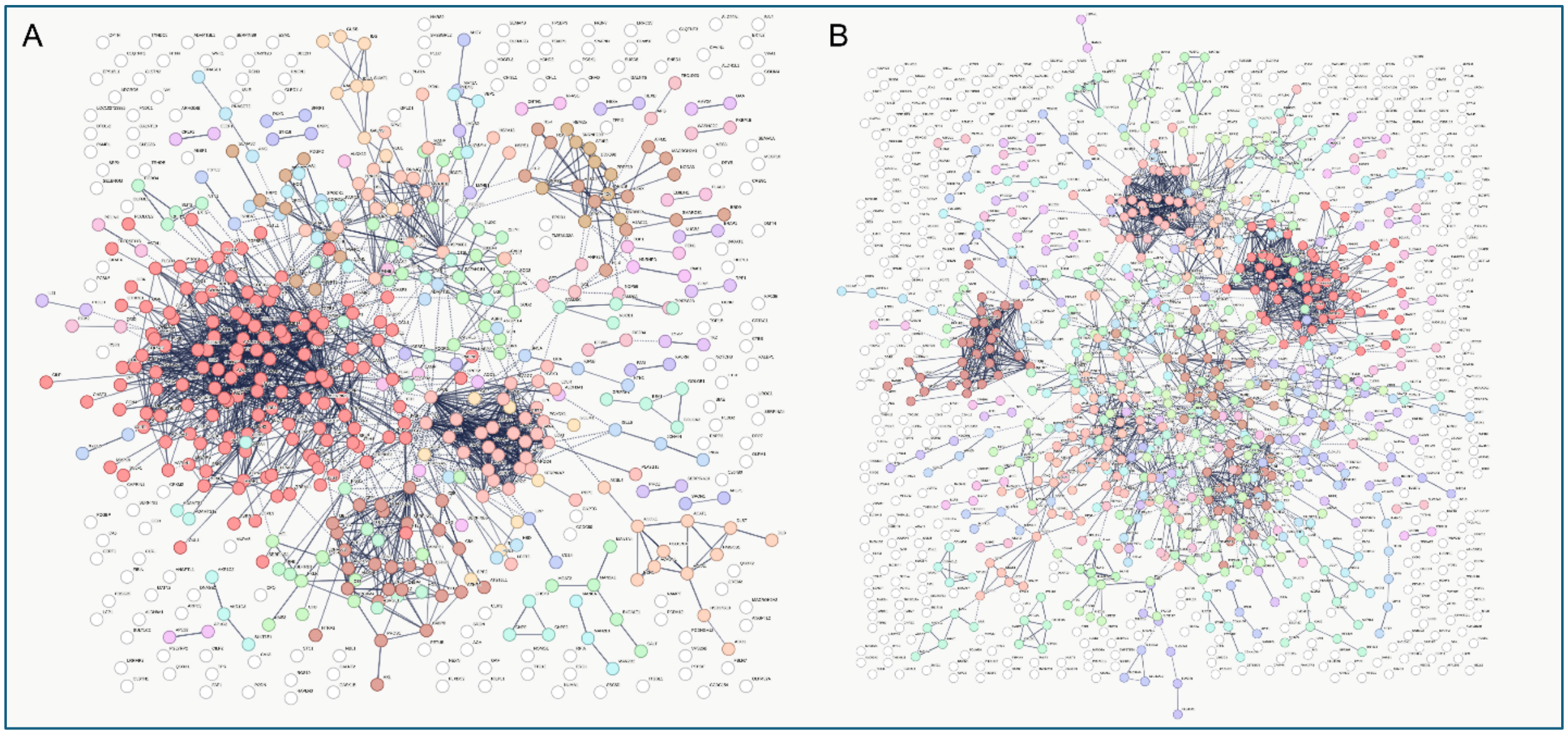

Supplementary Figures (S4): STRING protein–protein interaction networks for significantly upregulated (A) and downregulated (B) proteins. The parameters used for STRING analysis include confidence score = 0.7, FDR = 0.05, MCL inflation = 1.8 respectively. Dense subnetworks reveal enriched functional modules and coordinated regulation across phases, indicating biologically meaningful interaction hubs. This analysis identifies 71 upregulated and 148 downregulated functional clusters (≥5 proteins per cluster). Functional enrichment visualization showed that proteins enriched in late-phase sEVs were predominantly associated with extracellular matrix organization, adhesion signaling, vesicle biogenesis, and endolysosomal pathways, alongside immune and stress-responsive signaling modules, including RHO–ROCK, mTOR-associated signaling, and ER protein processing. Evidence of metabolic reprogramming was also apparent, with relative enrichment of lipid-handling and glycolytic pathways, whereas components of oxidative phosphorylation and several amino acid metabolic pathways were comparatively underrepresented. In contrast, proteins progressively depleted from sEVs over time were enriched for nuclear and biosynthetic processes, including RNA splicing, ribosome biogenesis, RNA polymerase II–mediated transcription, nucleosome organization, and core metabolic functions such as the TCA cycle and branched-chain amino acid degradation. The selective loss of these biosynthetic and transcriptional regulators is consistent with reduced proliferative capacity and translational output characteristic of aging and pre-senescent MSCs.

Supplementary Table (S4): list of founded proteins in phases intersections

	Phases intersection	Protein list	Number
Consistently expressed proteins	Exo-Up (2 vs 1) ^ (3 vs 1) ^ (3 vs 2)	LMNB1, DNJC3, AGRIN, FA20C, SRPX, NUCB1, FBLN4, TICN1, FBLN1, CO4A2, COGA1, COEA1, SVEP1, SNED1, OMD, AEBP1, HMCN1, CLC11, TENA, CPXM2, HIPL1	21
	Exo-Down (2 vs 1) ^ (3 vs 1) ^ (3 vs 2)	CLD11, LSAMP, RAI3, FLOT1, FLOT, S38A1	6
Transiently regulated proteins	Exo-Up (2 vs 1) ^ Exo-Down (3 vs 2)	STX16, THIL, CERT, RBP1, HNRPD, U520, GALT, NOP56, HMGB1, BKRB1, RBM25, TCOF, SRSF1, B4GA1, KPYR, ARK74, RET1, PGAM4, VGFR3, CC154, PTHR, AREG, HUTU, ST1E1, AL8A1, ST1C2, C3P1, DPYS, METK1, PLA1A, SEC20, XDH, ACES, NOGG, THYG, AL1L1, GSTA1; GSTA2; cRAP-GSTA1	37
	Exo-Up (3 vs 2) ^ Exo-Down (2 vs 1)	LRC15, CAH13, RTN1, SHH, ANGI	5

References:

Słotwiński M, Stepien E, Kyzioł A, Rawski M, Cryo-TEM and NTA Characterisation of Hyperglycemia-Induced Variability in Morphology and Size Distribution of EVs. Bio-Algorithms and Med-Systems. 2025; 21; 5.
